# Vigorous cool room treadmill training to improve walking ability in people with multiple sclerosis who use ambulatory assistive devices: a feasibility study

**DOI:** 10.1186/s12883-020-1611-0

**Published:** 2020-01-22

**Authors:** Augustine J. Devasahayam, Arthur R. Chaves, Wendy O. Lasisi, Marie E. Curtis, Katie P. Wadden, Liam P. Kelly, Ryan Pretty, Alice Chen, Elizabeth M. Wallack, Caitlin J. Newell, John B. Williams, Hannah Kenny, Matthew B. Downer, Jason McCarthy, Craig S. Moore, Michelle Ploughman

**Affiliations:** 1 Recovery & Performance Laboratory, Faculty of Medicine, Memorial University of Newfoundland, Rm 400, L.A. Miller Centre, 100 Forest Road, St. John’s, NL A1A 1E5 Canada; 2 Division of BioMedical Sciences, Faculty of Medicine, Memorial University of Newfoundland, Rm H4360, 300 Prince Philip Drive, St. John’s, NL A1B 3V6 Canada

**Keywords:** Progressive multiple sclerosis, Rehabilitation, Gait, Cooling, Neuroplasticity

## Abstract

**Background:**

Aerobic training has the potential to restore function, stimulate brain repair, and reduce inflammation in people with Multiple Sclerosis (MS). However, disability, fatigue, and heat sensitivity are major barriers to exercise for people with MS. We aimed to determine the feasibility of conducting vigorous harness-supported treadmill training in a room cooled to 16 °C (10 weeks; 3times/week) and examine the longer-term effects on markers of function, brain repair, and inflammation among those using ambulatory aids.

**Methods:**

Ten participants (9 females) aged 29 to 74 years with an Expanded Disability Status Scale ranging from 6 to 7 underwent training (40 to 65% heart rate reserve) starting at 80% self-selected walking speed. Feasibility of conducting vigorous training was assessed using a checklist, which included attendance rates, number of missed appointments, reasons for not attending, adverse events, safety hazards during training, reasons for dropout, tolerance to training load, subjective reporting of symptom worsening during and after exercise, and physiological responses to exercise. Functional outcomes were assessed before, after, and 3 months after training. Walking ability was measured using Timed 25 Foot Walk test and on an instrumented walkway at both fast and self-selected speeds. Fatigue was measured using fatigue/energy/vitality sub-scale of 36-Item Short-Form (SF-36) Health Survey, Fatigue Severity Scale, modified Fatigue Impact Scale. Aerobic fitness (maximal oxygen consumption) was measured using maximal graded exercise test (GXT). Quality-of-life was measured using SF-36 Health Survey. Serum levels of neurotrophin (brain-derived neurotrophic factor) and cytokine (interleukin-6) were assessed before and after GXT.

**Results:**

Eight of the ten participants completed training (attendance rates ≥ 80%). No adverse events were observed. Fast walking speed (cm/s), gait quality (double-support (%)) while walking at self-selected speed, fatigue (modified Fatigue Impact Scale), fitness (maximal workload achieved during GXT), and quality-of-life (physical functioning sub-scale of SF-36) improved significantly after training, and improvements were sustained after 3-months. Improvements in fitness (maximal respiratory exchange ratio and maximal oxygen consumption during GXT) were associated with increased brain-derived neurotrophic factor and decreased interleukin-6.

**Conclusion:**

Vigorous cool room training is feasible and can potentially improve walking, fatigue, fitness, and quality-of-life among people with moderate to severe MS-related disability.

**Trial registration:**

The study was approved by the Newfoundland and Labrador Health Research Ethics Board (reference number: 2018.088) on 11/07/2018 prior to the enrollment of first participant (retrospectively registered at ClinicalTrials.gov: NCT04066972. Registered on 26 August 2019.

## Background

Multiple Sclerosis (MS) is a chronic disease of the central nervous system (CNS), affecting approximately 2.3 million people worldwide [1]. MS is characterized by acute inflammatory episodes in the CNS, often transitioning to a progressive neurodegenerative phase [1]. About 80% of those who live with MS will develop the progressive form during their lifetime [1]. Cellular mechanisms contributing to neurodegeneration in MS include lack of trophic support to neurons and glia, chronic microglial activation, and mitochondrial injury induced by oxidative stress [2]. Several studies in animal models of MS suggest that exercise has direct protective and restorative effects by interacting with these mechanisms [3–5]. Evidence suggests that aerobic training promotes neuroplasticity by upregulating neurotrophins such as brain-derived neurotrophic factor (BDNF) and insulin-like growth factor (IGF-1) [6–9]. Further, aerobic exercise could have direct effects on the neuro-immune axis in MS [7, 10]. A systematic review of evidence suggested that aerobic training significantly altered peripheral levels of cytokines, interleukin (IL) 6, IL-10, interferon-gamma, and tumor necrosis factor-alpha [11]. Whether aerobic training has the potential to affect multiple underlying targets such as enhancing markers of neuroplasticity by upregulating neurotrophins and attenuating neural inflammation by altering levels of cytokines is not clear [12, 13]. Since aerobic exercise performed on a treadmill also provides a high volume of task-specific practice, aerobic treadmill training has the potential to improve walking ability, fitness, and quality of life [12–14].

Although aerobic training is a promising rehabilitative strategy for MS, aerobic exercise increases metabolic rate by 5 to 15 times above resting state and heat produced by contracting muscles elevates core body temperature, which in turn acts as a barrier to exercise participation [15–17]. Paroxysmal or fleeting MS symptoms, such as pins and needles that persist for few seconds to minutes, often occur as a result of a temperature-dependent conduction block in demyelinated axons, triggered by an increase in body temperature [18]. Impaired regulation of body temperature is a major barrier to exercise for people living with MS [19, 20]. In a cross sectional study, heat sensitive individuals with MS had simultaneous increase in core temperature and worsening of MS symptoms during aerobic exercise, when compared to resisted exercise [21]. Our previous research showed that cooling the exercise environment to 16 °C, mitigated exercise-induced losses in central drive among people with MS who reported having heat sensitivity [22]. Furthermore, there is a dearth of literature and rehabilitation options for individuals with higher levels of MS disability [12].

We aimed to determine the feasibility of conducting a vigorous aerobic walking training in a room cooled to 16 °C using bodyweight supported treadmill (BWST) for people with MS who were living with mitigable barriers to exercise participation such as MS-related walking disability (those who used ambulatory assistive devices, wheelchairs, and mobility scooters), fatigue, and heat sensitivity. We examined both the immediate and longer-term (at 3-month follow-up) impacts of training on walking speed, gait parameters, fatigue, aerobic fitness, and quality of life. We also examined in a preliminary way, whether the intervention would alter blood biomarkers of neuroprotection (BDNF) and inflammation (IL-6). BDNF and IL-6 were chosen as proxy indicators of neuroprotection and inflammation respectively because preliminary experiments showed that they were potential rehabilitative markers for people with MS having severe walking disability [23].

## Methods

### Design

This was a repeated measures feasibility study with a non-randomized single arm aimed to examine the feasibility and preliminary effects of the intervention. This study was approved by the Newfoundland and Labrador Health Research Ethics Board and registered in ClinicalTrials.gov database (NCT04066972). This study was conducted in accordance with the Tri-Council Policy Statement: Ethical Conduct for Research Involving Humans, 2014 and the principles outlined in the Declaration of Helsinki. This study conforms to the Consolidated Standards of Reporting Trials statement extension for feasibility studies [24].

### Sample size estimation

The target sample size for this study was estimated based on feasibility considerations. Our target sample size was between 10 and 15 participants, the size considered sufficient for studies evaluating feasibility issues in a single group of participants [25]. The secondary aim of this study was to detect walking speed differences measured by Timed 25 Foot Walk (T25FW) test (in seconds). To estimate the sample size required to assess preliminary effects of training in this study and to inform a future randomized controlled study, we used the data from a previous study [26, 27] where a training effect size of 0.994 was noted (decrease of values for T25FW from 10.9 ± 5.0 s at baseline to 6.8 ± 3.0 s after BWST training). Further, we considered data from Lo and Triche [26] for sample size calculation as their participant characteristics regarding walking difficulty matched our inclusion criteria. To detect a difference with 95% confidence and power of 80%, we required 11 participants for this study. As we expected a 20% rate of dropout or loss to follow–up during the study, we aimed to recruit 14 participants.

### Recruitment and screening

Participants were recruited from the local MS clinic and an outpatient rehabilitation service discharge database following written informed consent. The inclusion criteria were (a) clinically definite MS [28]; (b) relapse-free in the previous 3 months; (c) requiring ambulatory assistive devices (Expanded Disability Status Scale (EDSS)) score from 6.0 to 7.0) [29]; (d) negative Physical Activity Readiness Questionnaire (PAR-Q) screen for risk factors [30, 31]; and (e) greater than 6-weeks post Botulinum Toxin injection (if received) in lower extremity. The exclusion criteria were (a) pregnancy or intention of becoming pregnant; (b) finished a drug/device study in the last 30 days; (c) over 75 years of age; (d) unable to control bowel and bladder on physical exertion; (e) currently attending physical rehabilitation; and (f) having no difficulty walking in the community (self-selected walking speed > 120 cm/s). All participants were screened initially to determine whether they could participate in exercise using PAR-Q [30, 31]. The participants who failed the PAR-Q were referred to a physician for the PAR-Medical Examination (PAR-Med-X) [32]. Participants diagnosed with relapsing-remitting MS verified whether they had steadily increasing disability, without a clear recovery in the past year, to determine whether they were in transition to the progressive phase [33]. Finally, participants were asked to answer ‘Yes or No’ to the following questions: [1] ‘Do you experience fatigue?’ and [2] ‘Are you sensitive to heat?’

### Outcome measures

#### Feasibility

Feasibility of conducting vigorous training in participants with barriers to exercise was assessed using a checklist that included attendance rates, number of missed appointments, reasons for not attending, adverse events (MS relapse, syncope, or medical emergencies), safety hazards during training (difficulty getting on and off treadmill, difficulty adjusting to changes in treadmill speed and inclination, difficulty switching between sitting and standing positions), reasons for dropout, tolerance to training load (degree of body weight support required during exercise, number of breaks taken during exercise, minutes of exercise), subjective reporting of symptom worsening during and after exercise, and physiological responses to exercise (tympanic temperature, heart rate, fatigue on a visual analog scale, and mean arterial pressure measured before and after exercise).

#### Walking speed

Fast walking speed was assessed on a path clear of obstacles in a quiet, private environment using two methods, (i) T25FW test [34–36], and (ii) on a 4″ X 14″ computerized Protokinetics Zeno™ walkway in order to measure spatiotemporal parameters of fast walking [37]. Participants were also instructed to walk two laps on the walkway at self-selected walking speed to measure speed and spatiotemporal parameters. For all walking assessments, participants were provided with standardized instructions and used their ambulatory devices. If the participants required additional assistance while walking, they were assisted using a gait belt by a member of the research team, who was a physiotherapist. Gait parameters (stance phase (%), swing phase (%), double support phase (%), and walking speed (cm/s)) were extracted from the walkway as previously described [38].

#### Fatigue

Fatigue was assessed using three methods: (a) The fatigue/energy/vitality sub-scale of 36-Item Short-Form (SF-36) Health Survey measured the extent of fatigue [39–41], (b) The Fatigue Severity Scale (FSS) measured the intensity of fatigue [42] and (c) the modified Fatigue Impact Scale (mFIS) measured the impact of fatigue on everyday life [43–45].

#### Aerobic fitness

Fitness was assessed using maximal GXT on a seated recumbent stepper [46]. All participants were asked to exercise at increasingly difficult levels while wearing a facemask to measure how much oxygen they consumed (Moxus Metabolic Systems; AEI Technologies, Inc.). A heart rate monitor was placed on their chest to measure maximal heart rate achieved during GXT. Participants were verbally encouraged to exercise as long as they could, and the workload was increased in ~ 20-watt increments every 2 min, starting from load level 3 (21 watts) until exhaustion [46]. Participants were considered to have attained maximal oxygen consumption (V̇O_2_) if at least two of the following criteria were met: (a) V̇O_2_ plateau (no increase in V̇O_2_ by 150 mL/min despite increasing workload) [46, 47], (b) respiratory exchange ratio > 1.10 [47], (c) > 90% age-predicted maximal heart rate [47], and/or (d) > 8/10 rate of perceived exertion [47].

#### Quality of life

Health-related quality of life was assessed using SF-36 Health Survey, which consisted of nine domains including physical functioning, role limitations due to physical health, role limitations due to emotional problems, mental health/emotional well-being, social functioning, bodily pain, fatigue/energy/vitality, general health perceptions, and health compared to last year [39–41].

### Serum analysis

Blood was collected from the median cubital vein at three testing time points (pre, post, and follow-up) immediately before and after GXT in two 5 mL serum vacutainers [48]. The samples were left to clot for 30–60 min, centrifuged at 2200 g for 10 min, and the collected serum was stored frozen at − 80 °C until assayed. Serum levels of neurotrophin (BDNF) and cytokine (IL-6) were measured using ELISA kits for human BDNF (R&D Systems Inc. Minneapolis, Minnesota, USA) and IL-6 (BD Biosciences, San Diego, California, USA) as per manufacturer’s instructions.

### Intervention

All participants underwent a personalized, progressively intense, moderate to vigorous intensity (40–65% heart rate reserve (HRR) [49]) training for ten weeks (3x/week) in a temperature-controlled room (16 °C) starting at 80% self-selected walking speed on a BWST equipped with safety straps to prevent falls. Exercise intensity was estimated using resting and maximal heart rates measured before and during GXT respectively at baseline (Exercise heart rate = % target intensity (maximal heart rate – resting heart rate) + resting heart rate) [47, 50]. Each training session lasted up to 40 min, including 5 min of warm-up and cool-down. A gradual progression of workload was undertaken to minimize muscle injury [51], starting with moderate intensity (40% HRR) at 80% self-selected speed, and progressing to vigorous intensity (65% HRR) at gradually increasing walking speed as tolerated [49], with simultaneous reduction of bodyweight support provided on the treadmill from 10 to 0% and increase of treadmill incline from 1 to 10%. For individuals with severe walking impairment, manual support was provided to advance the weaker lower extremity as required.

### Data analysis

Variables were assessed if they met assumptions of non-parametric statistics. Statistical analyses (Friedman rank test followed by Wilcoxon matched-pairs signed-rank tests with Bonferroni alpha correction (0.05/3 = 0.01)) were then conducted to determine the effects of cool room BWST training. Missing data were not imputed but were excluded pairwise due to small sample size and its concurrent higher variance. The relationships between the outcome measures were estimated by Spearman’s rank correlation coefficient (r_s_). When multiple correlations were conducted, a post-hoc correction was performed using the Bonferroni method [52, 53]. Clinically meaningful changes, both individual as well as a group, were determined for participants who attended all three testing time points using cut-off values published previously in the literature.

## Results

### Feasibility of recruitment, attendance, and retention

#### Recruitment

Thirty-seven MS patients were contacted to determine their willingness to participate. Thirteen MS patients did not meet eligibility criteria, seven declined to participate, and seven were not contactable (see Flow Chart in Additional file 1). Out of 10 MS patients who agreed to participate, eight passed the PAR-Q, and two passed PAR-Med-X, and were thus enrolled (*n* = 10) in the study (Table 1). Recruitment was stopped prior to reaching enrollment goal (*n* = 11) due to slow accrual and difficulty finding patients who were willing to participate in the training program. All participants (n = 10) identified themselves as having fatigue and sensitivity to heat. Ten participants (9 females), aged 29 to 74 years, with EDSS ranging from 6.0 to 7.0 completed the baseline assessments following which, two dropped out of the study (after completing 2 and 7 sessions respectively), and eight participants continued to participate in the exercise training sessions (range, 24 to 30 sessions) (Table 1). Eight participants (7 females) completed the 10-week exercise training and completed the assessments immediately after the training program. Three months after exercise training, seven participants (6 females) returned to complete the follow-up assessments.

**Table 1:** Attendance characteristics

Participant	Total number of sessions attended	Attendance rate	Total number of missed appointments	Discontinued intervention (Yes/No)
1	26	86.67	3	No
2	24	80.00	3	No
3	30	100.00	1	No
4	26	86.67	6	No
5	26	86.67	5	No
6	7	23.33	7	Yes
7	30	100.00	1	No
8	25	83.33	5	No
9	28	93.33	3	No
10	2	6.67	1	Yes

#### Attendance rates and reasons for missed appointments

The attendance rates ranged from 80 to 100% among those who completed exercise training and the total number of missed appointments ranged from 1 to 6 per participant (Table 1). The reasons for missing appointments were feeling tired or unwell (*n* = 15), transportation issues (*n* = 5), having medical appointments (*n* = 4), personal scheduling conflict (n = 4), leg pain and stiffness (*n* = 3), inclement weather (n = 3), recent fall (*n* = 2), and forgot appointment (n = 1). Participants rescheduled the missed appointments and continued to participate in the exercise sessions (Table 1).

#### Baseline characteristics

On average, the participants were 53.2 years of age (± 15.6) and had a body mass index of 28.2(± 6.6) (Table 2). Four had confirmed diagnosis of progressive MS and six were in transition from relapsing-remitting to progressive phase (Table 2) [33]. Participants used either unilateral (*n* = 4) or bilateral (*n* = 6) support during ambulation (Table 2). On average, self-selected walking speed was 57.8( ± 31.3) cm/s, and fast walking speed was 85.8( ± 54.4) cm/s. None of the participants required additional assistance from the physiotherapist during overground walking speed assessments.

**Table 2:** Participant characteristics

N	EDSS	Type of MS	Sex	Age	Years since MS Diagnosis	BMI	Ambulatory assistive device used (indoor/outdoor)	Fast walking speed (in cm/s)	Self-selected walking speed (in cm/s)
1	7.0	PPMS	F	57	10	38.20	Rollator walker/Motorized wheelchair	31.29	24.89
2	7.0	SPMS	F	58	33	30.90	Rollator walker/Motorized scooter	26.74	16.99
3	7.0	PPMS	M	42	19	25.60	Rollator walker/Wheelchair	82.42	47.22
4	6.5	SPMS^a^	F	50	28	17.90	Cane	204.95	102.06
5	7.0	SPMS^a^	F	38	19	32.30	2 Canes/Motorized scooter	98.15	83.77
6	7.0	SPMS^a^	F	42	8	31.50	Rollator walker	84.02	63.04
7	6.0	SPMS^a^	F	72	18	20.30	Cane	122.21	92.90
8	7.0	PPMS	F	74	10	32.30	Rollator walker/Wheelchair	20.72	14.15
9	6.0	SPMS^a^	F	29	2	31.90	Cane	85.33	66.52
10	6.0	SPMS^a^	F	70	29	21.30	Cane	102.50	66.74

*N* Participant number, *EDSS* Expanded disability status scale, *MS* Multiple sclerosis, *BMI* Body mass index, *cm* Centimeter, *sec* Second, *PPMS* Primary progressive MS, *SPMS* Secondary progressive MS, *F* Female, *M* Male; ^a^participants in transition from relapsing-remitting to progressive phase of MS who reported steadily increasing disability, without a clear recovery in the past one year;

### Feasibility of intervention

#### Adverse events, safety, and dropouts

The intervention was laboratory-based in a rehabilitation hospital setting; therefore, the researchers relied on physicians-on-call for emergencies. No adverse events (MS relapse, syncope, or medical emergencies) occurred during assessments and training sessions. One participant required electrocardiograph monitoring by the physician during GXT due to a history of arrhythmia. The GXT was terminated due to high systolic blood pressure (> 220 mmHg); however, the participant was admitted into the study after clearance from the physician. Participants wore a safety harness during all training sessions and no safety hazards were identified. Two participants discontinued intervention and one participant was lost to follow up (see Additional file 1).

#### Training load and tolerance

**Fig. 1:**
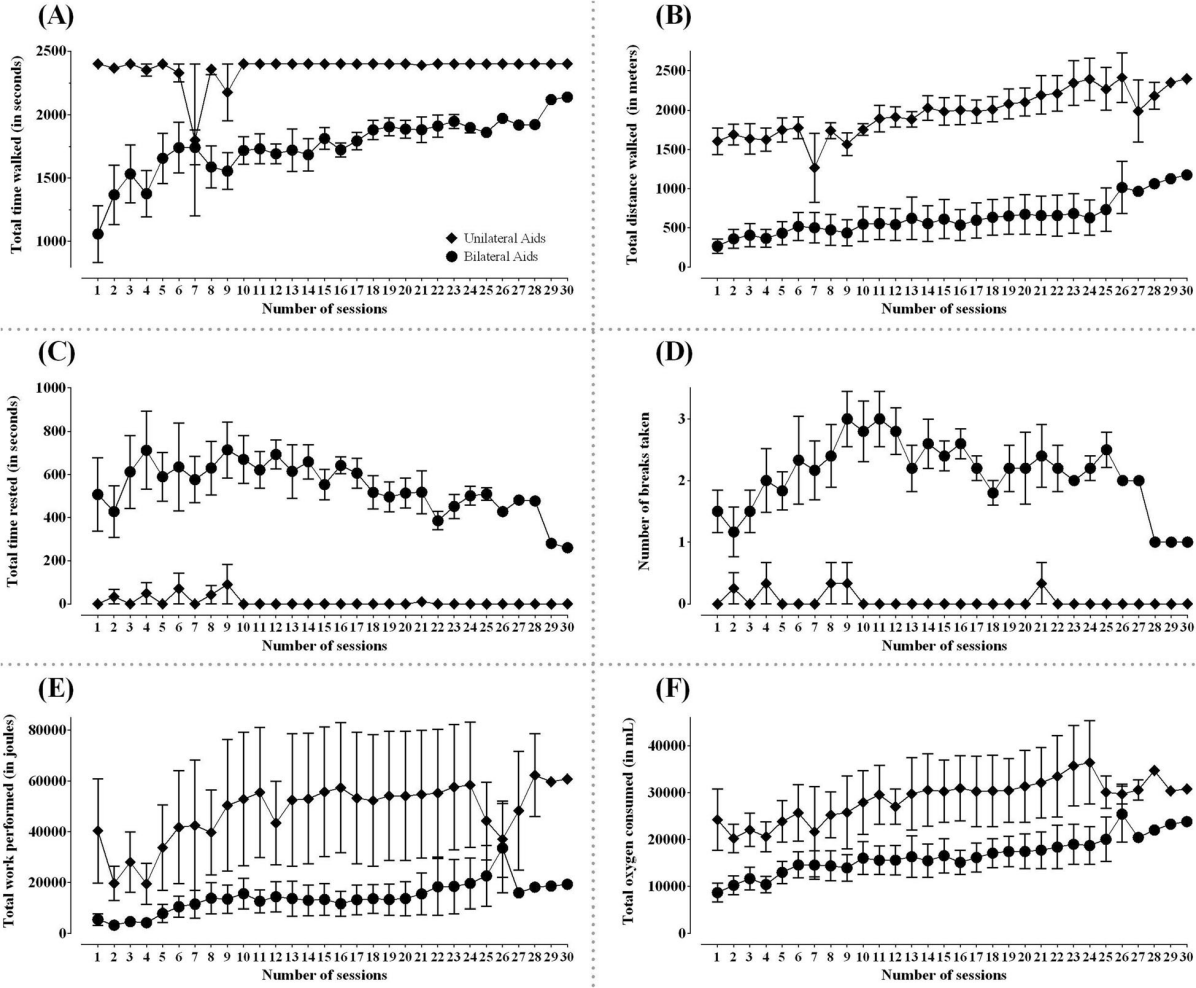
Safety and feasibility of the intervention. Data are presented as means and standard errors for thirty training sessions separately in participants who used unilateral and bilateral walking aids. **a**: total time walked (in seconds); **b**: total distance walked (in meters); **c**: total time rested (in seconds); **d**: number of breaks taken; **e**: total work performed (in joules); **f**: total oxygen consumed (in milliliters); Solid diamonds: unilateral walking aid users; Solid circles: bilateral walking aid users

All participants were able to perform progressively intense BWST training from moderate to vigorous intensity (40–65% HRR) [49], however participants did not have significant change in resting heart rate after training (*p* = 0.29). Eight out of 10 participants were able to walk on the treadmill at 80% of their self-selected overground walking speed from the first exercise session onwards. One participant was able to start training at 60% and another at 40% of their respective self-selected overground walking speeds. All participants, but one, were able to walk on the treadmill with 10% body weight support from the first exercise session. Three participants were able to completely wean off to 0% body weight support over ten weeks. Three participants required manual assistance to advance their lower extremity during initial treadmill training sessions, which was weaned off gradually. The total time walked, and distance covered progressively increased while the total time required to rest decreased (Figs. 1a-d). The participants were advised to take breaks in either sitting or standing position on the treadmill as required during training sessions (Fig. 1d). There was an overall increase in workload performed and oxygen consumed in both unilateral and bilateral walking aid users (Figs. 1e and f).

#### Subjective reporting of symptoms and physiological response to exercise

**Fig. 2:**
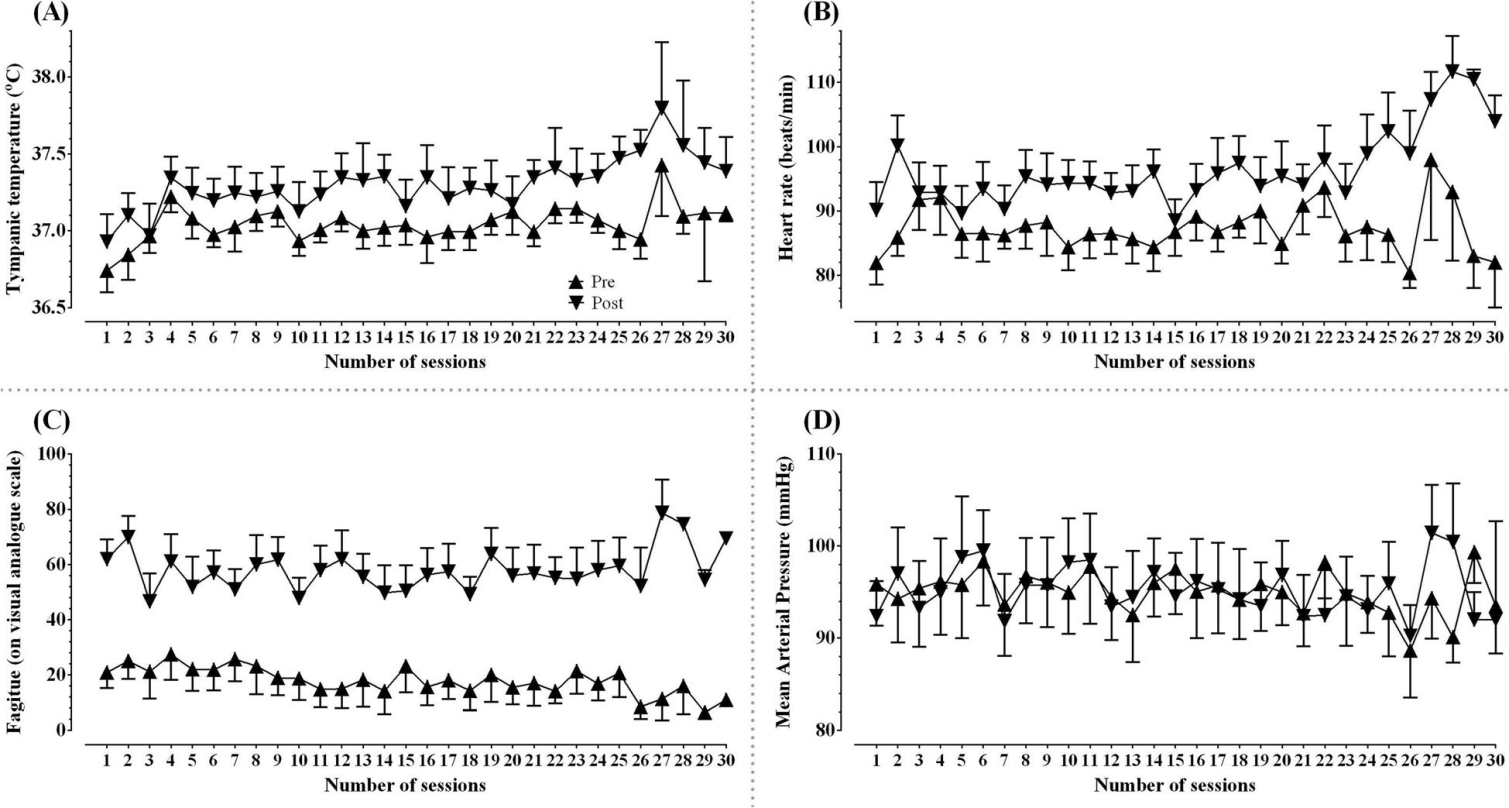
Physiological responses to a temperature-controlled environment. Data collected immediately before and after training sessions are presented as means and standard errors for thirty training sessions separately. **a**: tympanic temperature (°C); **b**: heart rate (beats per minute); **c**: fatigue (on a visual analog scale); **d**: mean arterial pressure (millimeters of mercury); Upright triangles: pre-exercise; Inverted triangles: post-exercise

All participants were able to tolerate the cool room training with the air-conditioning set at 16 °C. Two participants reported having mild symptoms, such as pins and needles sensations, that were fleeting for a few seconds or minutes during training sessions. Two participants reported having weak legs while walking on the treadmill. One participant complained of shoulder ache after bearing body weight through arms and requested greater body weight support. One participant had leg pain that resulted in the termination of one of the training sessions. None of the participants reported exacerbation of MS symptoms, such as the occurrence of motor weakness, ataxia of a limb, or any other MS symptoms, that lasted more than 24 h after training sessions [54, 55]. Tympanic temperature, heart rate, and fatigue increased with exercise, while mean arterial pressure remained stable (Fig. 2a-d).

### Secondary outcomes

#### Walking

##### Fast walking speed

**Fig. 3:**
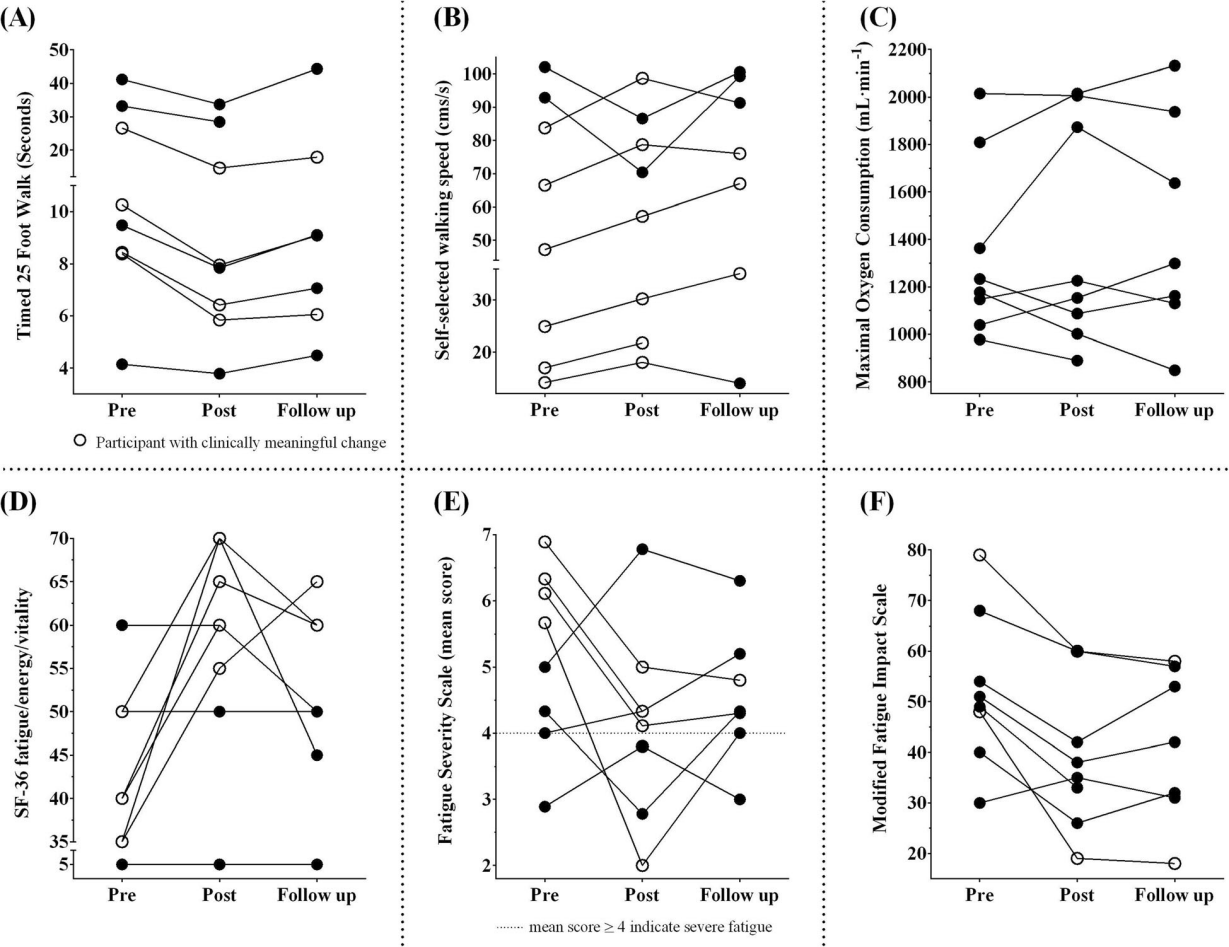
Effects of vigorous aerobic cool room training in MS. Data are presented as individual values. **a**: walk time measured using timed 25 ft walk test (in seconds); **b**: self-selected walking speed (in centimeters per second); **c**: maximal oxygen consumption (in milliliter per minute); **d**: fatigue measured using short-form 36 fatigue/energy/vitality subscale; **e**: fatigue measured using fatigue severity scale (mean scores); **f**: fatigue measured using modified fatigue impact scale; open circles: participants with clinically meaningful change

We tested fast walking speed using two methods, [1] T25FW test (in seconds) and [2] on an instrumented walkway (cm/s). In terms of the T25FW test, following ten weeks of training, participants walked 1.4 times faster, but values returned to pre levels at follow up (Table 3). Four out of 8 participants made a clinically meaningful change (≥ 20%) after training (Fig. 3a) [56–58].

**Table 3:** Effects of vigorous cool room training on walking, fatigue, and fitness

Variable	Pre	Post	Follow-up	Test statistic	p	Post-hoc (p-adj)
	M (SD)	M (SD)	M (SD)			
Walking						
T25FW (s)	15.51 (13.41)	11.45 (10.38)	14.00 (14.07)	11.143	0.004*	t_1–2_ = 0.012*
						t_1–3_ = 0.237
						t_2–3_ = 0.018*
Fast walking speed (cm/s)	92.15 (61.39)	106.44 (65.74)	103.61 (68.34)	10.286	0.006*	t_1–2=_0.012*
						t_1–3_ = 0.043*
						t_2–3_ = 0.237
Self-selected walking speed (cm/s)	61.64 (34.01)	62.86 (29.62)	69.05 (33.27)	2.000	0.368	t_1–2_ = 0.674
						t_1–3_ = 0.063
						t_2–3_ = 0.237
Fatigue						
SF-36 fatigue/energy/vitality	39.29 (17.66)	53.57 (22.68)	47.86 (20.18)	4.105	0.128	t_1–2_ = 0.039*
						t_1–3_ = 0.225
						t_2–3_ = 0.216
Fatigue Severity Scale (mean score)	4.98 (1.36)	4.11 (1.54)	4.56 (1.03)	0.222	0.895	t_1–2_ = 0.123
						t_1–3_ = 0.345
						t_2–3_ = 0.398
Modified Fatigue Impact Scale (total score)	52.86 (16.48)	40.00 (15.65)	41.57 (15.26)	5.429	0.066	t_1–2_ = 0.017*
						t_1–3_ = 0.034*
						t_2–3_ = 0.553
Modified Fatigue Impact Scale (Physical)	25.71 (4.31)	19.29 (6.37)	19.29 (5.50)	8.000	0.018*	t_1–2_ = 0.024*
						t_1–3_ = 0.018*
						t_2–3_ = 0.932
Modified Fatigue Impact Scale (Cognitive)	22.29 (12.91)	16.29 (8.32)	18.57 (9.57)	6.000	0.050	t_1–2_ = 0.079
						t_1–3_ = 0.089
						t_2–3_ = 0.172
Modified Fatigue Impact Scale (Psychosocial)	4.86 (1.68)	4.43 (2.07)	3.71 (1.70)	0.737	0.692	t_1–2_ = 0.131
						t_1–3_ = 0.276
						t_2–3_ = 0.673
Aerobic Fitness						
V̇O_2_ max (mL/min/kg)	18.30 (5.37)	19.50 (5.85)	19.77 (7.23)	0.857	0.651	t_1–2_ = 0.484
						t_1–3_ = 0.398
						t_2–3_ = 0.735
HR max (beats/min)	147.00 (23.68)	149.71 (23.09)	153.14 (30.28)	1.680	0.432	t_1–2_ = 0.078
						t_1–3_ = 0.237
						t_2–3_ = 0.611
Maximum Workload (watts)	110.43 (42.91)	123.86 (46.39)	123.43 (46.54)	7.714	0.021*	t_1–2_ = 0.012*
						t_1–3_ = 0.043*
						t_2–3_ = 0.866
Oxygen Uptake Efficiency Slope (V̇E_log10_/V̇O_2_mL/min)	1466.19 (412.62)	1633.73 (474.79)	1588.36 (502.25)	2.000	0.368	t_1–2_ = 0.123
						t_1–3_ = 0.237
						t_2–3_ = 1.000
Oxygen Uptake Efficiency Slope (V̇E_log10_/V̇O_2_mL/min/kg)	18.76 (4.21)	21.14 (4.71)	21.30 (6.81)	5.429	0.066	t_1–2_ = 0.049*
						t_1–3_ = 0.091
						t_2–3_ = 0.735

*M* Mean, *SD* Standard deviation p: significance; p-adj: adjusted *p* value; *p < 0.05; *T25FW* Timed 25-Foot Walk, *s* Second, *cm* centimeter, *SF-36* 36-Item Short Form Health Survey; *mL* Milli-liter, *min* Minute, *kg* Kilogram, *V̇E* Ventilation, *V̇O*_*2*_ Oxygen consumption;

In regards to fast walking speed measured on the instrumented walkway (cm/s), speed increased by 15.5%, which was sustained at follow up compared to pre assessment (Table 3). Furthermore, gait quality (duration of stance phase (%), swing phase (%), and total double support phase (%)) during fast walking improved at post (*p* values, 0.025, 0.025 and 0.017 respectively), but values returned to pre levels at follow up (p values, 0.13, 0.13 and 0.13).

##### Self-selected walking speed

There was no significant change in self-selected walking speed (cm/s) (measured on an instrumented walkway) at both post and follow up (Table 3). However, 6 out of 8 participants made a clinically meaningful change of more than 12% beyond the benchmark accepted for walking assessments in MS (Fig. 3b) [59, 60].

There was no significant change in stance and swing phases (%) while walking at self-selected speed (p values, 0.09 and 0.09 respectively), however total double support phase (%) was significantly reduced at post compared to pre (*p* = 0.036). Duration of the stance phase (%), swing phase (%), and total double support phase (%) while walking at self-selected speed improved significantly at follow up compared to pre (p values, 0.018, 0.018, and 0.018).

#### Fatigue

Participants rated three aspects of fatigue, [1] present level of energy (fatigue/energy/vitality sub-scale of SF-36 Health Survey), [2] severity of fatigue (FSS), and [3] impact of fatigue on everyday life (mFIS). Participants reported improved fatigue (36.4% or 14.3 point increase in energy levels on fatigue/energy/vitality sub-scale of SF-36 Health Survey) at post, which returned to pre levels at follow up (8.6 point increase from pre) (Table 3) (Fig. 3d). However, 5 out of 8 participants made a minimally important improvement of 11.3 or more points at post, of whom 3 participants sustained the improvements at follow up (Fig. 3d) [61].

Severity of fatigue reported on FSS (mean score) was not significantly different at post or at follow up compared to pre (Table 3). However, 4 out of 8 participants achieved a change of 1.9 or more points on mean FSS scores at post, a minimal detectable clinically meaningful change for people with MS (Fig. 3e) [43].

Impact of fatigue reported on mFIS was significantly less at post, which was sustained at follow up compared to pre (Table 3). However, only 1 out of 8 participants had a clinically meaningful change beyond the accepted benchmark of 20.2 points at post, and two at follow up (Fig. 3f) [43].

#### Aerobic fitness

There was no statistically significant change in maximal V̇O_2_ and maximal heart rate achieved during GXT at post compared to pre (Table 3) (Fig. 3c). However, the participants were able to achieve a greater workload during GXT at both post and follow up compared to pre values (Table 3). The oxygen uptake efficiency slope, a measure of the cardiorespiratory reserve, significantly increased at post, which was sustained during follow up (Table 3) [62, 63].

In terms of indicators of achievement of a maximal GXT, four out of 10 participants achieved two or more criteria for test termination at pre, 3 out of 8 at post, and 3 out of 7 at follow up [46, 47]. The maximal respiratory exchange ratio ranged from 0.84 to 1.28 (1.07 ± 0.15) at pre, 0.93 to 1.24 (1.07 ± 0.12) at post, and 0.90 to 1.21 (1.07 ± 0.11) at follow up, in which five out of 10 participants achieved respiratory exchange ratio more than 1.1 at pre, 4 out of 8 at post, and 3 out of 7 at follow up. The maximal age-predicted heart rate achieved by participants ranged from 68.1 to 101.4% (88.1 ± 11.9%) at pre, 68.1 to 104.1% (88.8 ± 12.2%) at post, and 69.1 to 114.2% (91.6 ± 16.9%) at follow up, in which five out of 10 participants achieved more than 90% of their age-predicted maximal heart rate at pre, 4 out of 8 at post, and 4 out of 7 at follow up. Borg’s rating of perceived exertion reported at the end of GXT ranged from 6.0 to 10.0 (9.2 ± 1.5) at pre, 7.0 to 10.0 (9.5 ± 1.1) at post, and 7.0 to 10.0 (9.6 ± 1.1) at follow up, in which eight out of 10 participants rated more than 8.0 on Borg’s rate of perceived exertion at pre, 7 out of 8 at post, and 6 out 7 at follow up. All participants reported performing GXT to their maximal volitional exhaustion at all testing time points, except for two participants who reported that they could have pushed themselves more during GXT performed at post-training.

#### Quality of life

There was a clinically meaningful improvement in the quality of life in all SF-36 domains (i.e., more than a 3-point increase in all SF-36 domains separately at post compared to pre), except social functioning (1.8 point increase) (Tables 3 and 4) [64, 65]. Physical functioning significantly improved at both post and follow up compared to pre (Table 4). Perception about overall health (compared to last year) and bodily pain significantly improved at post, but not at follow up (Table 4). Although not statistically significant, we noted clinically meaningful (≥ 3-point increase) improvements reported at post on the SF-36 subscales - role limitations due to physical health, role limitations due to emotional problems, mental health/emotional well-being, and general health perceptions. We also noted clinically meaningful (≥ 3-point increase) improvements sustained until follow up compared to pre, in bodily pain, general health perceptions, and health compared to last year.

**Table 4:** Effects of vigorous cool room training on quality of life

Variable	Pre	Post	Follow-up	Test statistic	p	Post-hoc (p-adj)
	M (SD)	M (SD)	M (SD)			
Quality of life						
SF-36 physical functioning	28.57 (21.55)	36.43 (24.10)	41.43 (28.24)	8.083	0.018*	t_1–2_ = 0.038*
						t_1–3_ = 0.027*
						t_2–3_ = 0.395
SF-36 role limitations due to physical health	32.14 (42.61)	35.71 (37.80)	32.14 (37.40)	0.105	0.949	t_1–2_ = 0.414
						t_1–3_ = 1.000
						t_2–3_ = 1.000
SF-36 role limitations due to emotional problems	76.19 (41.79)	80.94 (32.55)	71.43 (48.80)	0.667	0.717	t_1–2_ = 0.317
						t_1–3_ = 0.655
						t_2–3_ = 0.593
SF-36 mental health/emotional well-being	72.57 (15.57)	81.71 (14.76)	74.86 (16.28)	5.840	0.054	t_1–2_ = 0.067
						t_1–3_ = 0.336
						t_2–3_ = 0.112
SF-36 social functioning	73.21 (11.25)	75.00 (28.87)	69.64 (25.88)	0.095	0.953	t_1–2_ = 0.746
						t_1–3_ = 0.516
						t_2–3_ = 0.705
SF-36 bodily pain	46.79 (12.22)	69.29 (16.50)	63.93 (19.73)	6.080	0.048*	t_1–2_ = 0.018*
						t_1–3_ = 0.093
						t_2–3_ = 0.674
SF-36 general health perceptions	41.43 (17.49)	52.14 (21.19)	47.14 (19.12)	1.923	0.382	t_1–2_ = 0.105
						t_1–3_ = 0.340
						t_2–3_ = 0.236
SF-36 health compared to last year	46.43 (29.73)	67.86 (18.90)	71.43 (22.49)	7.176	0.028*	t_1–2_ = 0.024*
						t_1–3_ = 0.066
						t_2–3_ = 0.564

*M* Mean, *SD* Standard deviation; p: significance; p-adj: adjusted *p* value; *p < 0.05; *SF*-36 36-Item Short Form Health Survey;

#### Blood biomarkers

We were unable to draw blood samples from three participants on 6 out of 52 occasions. All serum BDNF levels were within the detectable ranges. Serum IL-6 levels were not detectable in seven participants on 22 out of 46 occasions.

##### Neurotrophins

**Fig. 4:**
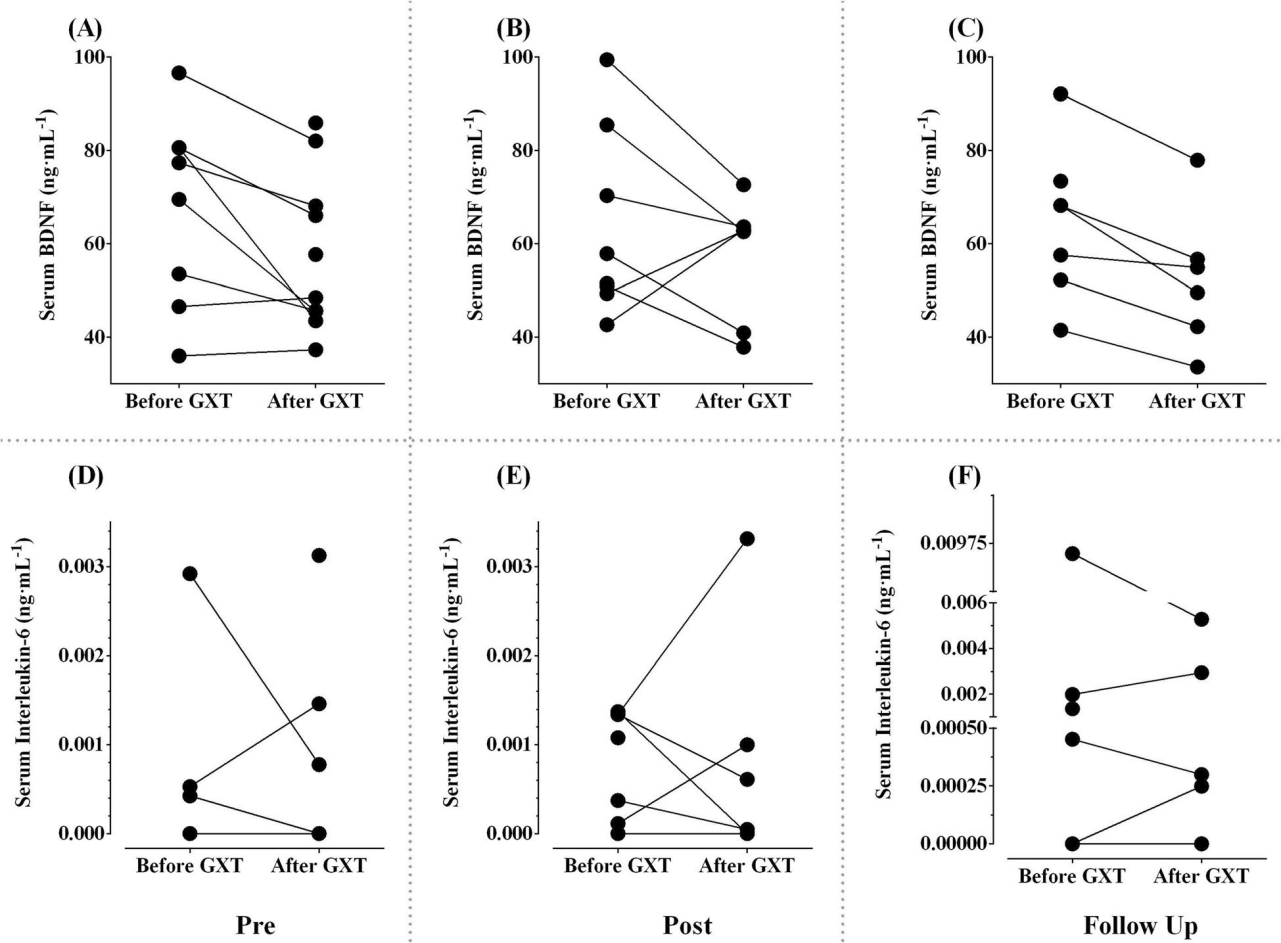
Blood biomarker responses to graded exercise test. Data are presented as individual values. **a**, **b**, **c**: serum brain-derived neurotrophic factor (in nanogram per milliliter) at pre, post, and follow up respectively; **d**, **e**, **f**: serum interleukin-6 (in nanogram per milliliter) at pre, post, and follow up respectively

In terms of serum BDNF, there were no significant differences in resting and exercise-induced levels (After minus Before GXT) measured at pre (Fig. 4a), post (Fig. 4b), and at follow up (Fig. 4c) (*p* values, 0.22 and 1.0 respectively) (Table 5). There was a significant decrease in serum BDNF after GXT compared to before GXT levels, both at pre (Fig. 4a) and follow up (Fig. 4c) (p values, 0.036 and 0.028 respectively), but not at post-training (*p* = 0.31) (Fig. 4b).

**Table 5:** Effects of vigorous cool room training on blood biomarkers

Variable	Pre	Post	Follow-up	Test statistic	p	Post-hoc (p-adj)
	M (SD)	M (SD)	M (SD)			
Biomarkers						
Brain derived neurotrophic factor at rest (ng/mL)	67.62 (20.43)	63.46 (19.97)	64.76 (16.31)	3.000	0.223	t_1–2_ = 0.237
						t_1–3_ = 0.345
						t_2–3_ = 0.499
Interleukin-6 at rest (ng/mL)	0.0005 (0.0010)	0.0007 (0.0006)	0.0019 (0.0035)	2.533	0.282	t_1–2_ = 0.686
						t_1–3_ = 0.109
						t_2–3_ = 0.225

*M* Mean, *SD* Standard deviation; p: significance; p-adj: adjusted *p* value; *p < 0.05; *ng* Nanogram, *mL*, Milli-liter;

##### Cytokines

In terms of serum IL-6, there were no significant differences in resting and exercise-induced levels (After minus Before GXT) measured at pre (Fig. 4d), post (Fig. 4e), and at follow up (Fig. 4f) (p values, 0.28 and 0.37) (Table 5). There was no significant change in serum IL-6 after GXT compared to before GXT levels, at pre (Fig. 4d), post (Fig. 4e), and follow up (Fig. 4f) (*p* values, 0.59, 0.90, and 1.0 respectively).

#### Relationship between outcomes

**Fig. 5:**
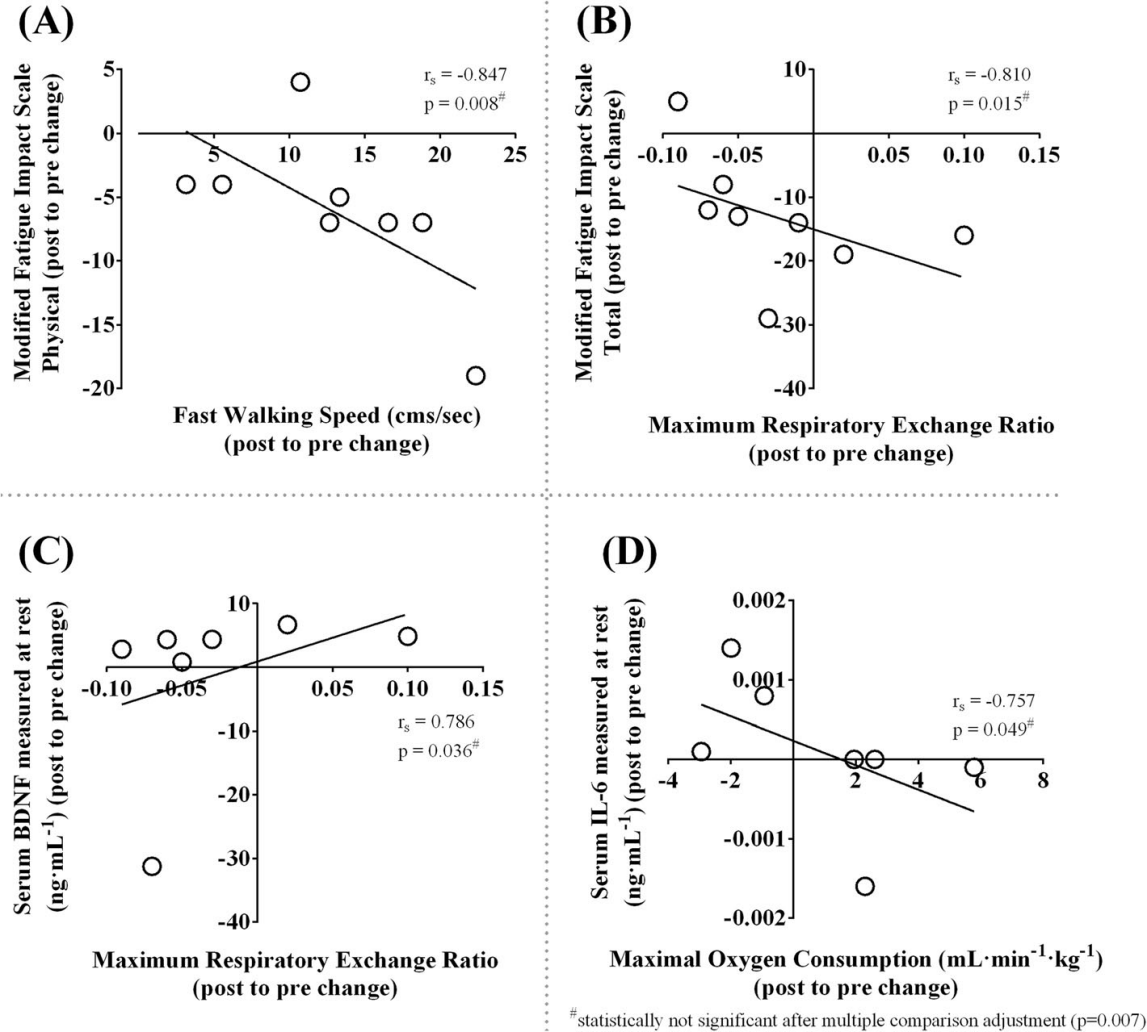
Relationship between outcomes. **a**: correlation between modified fatigue impact scale physical subcomponent change score and fast walking speed change score (*r*_*s*_ = − 0.847, *p* = 0.008); **b**: correlation between modified fatigue impact scale total change score and maximal respiratory exchange ratio change score (*r*_*s*_ = − 0.810, *p* = 0.015); **c**: correlation between resting serum brain-derived neurotrophic factor change score and maximal respiratory exchange ratio change score (*r*_*s*_ = 0.786, *p* = 0.036); **d**: correlation between resting serum interleukin-6 change score and maximal oxygen consumption change score (*r*_*s*_ = − 0.757, *p* = 0.049). r_s_, Spearman correlation coefficient; p, significance

The improvement in fast walking speed was associated with reduced fatigue measured using physical subcomponent score of mFIS (Spearman’s rank correlation coefficient, r_s_ = − 0.847, *p* = 0.008) (Fig. 5a). The improvement in fatigue measured using total mFIS score was related to higher maximal respiratory exchange ratio achieved during GXT (r_s_ = − 0.810, *p* = 0.015) (Fig. 5). The improvement in maximal respiratory exchange ratio achieved during GXT was associated with an increase in resting serum BDNF (r_s_ = 0.786, *p* = 0.036) (Fig. 5c). The improvement in fitness measured using maximal V̇O_2_ was associated with a decrease in resting serum IL-6 (r_s_ = − 0.757, *p* = 0.049) (Fig. 5d). However, after correcting for multiple correlations (0.05/7 = 0.007) [52, 53], none of the relationships were statistically significant.

## Discussion

We tested a novel cool room intensive treadmill training for people with moderate to severe walking disability in the progressive phase of MS or transitioning to the progressive phase. We found that this intervention was feasible, and most participants achieved clinically meaningful improvements in walking, fatigue, fitness, and quality of life.

### Feasibility of vigorous cool room training in MS

In 1890, Uhthoff [66] reported that patients with MS had exercise-induced amblyopia, a phenomenon later discovered to be due to an increase in body temperature [18]. Nearly 60 years later, Watson [67] demonstrated several positive effects of cold exposure in patients with MS, including improvements in pain, sensation, vision, motor control, and mood. Since then, data from controlled experimental studies suggested that both pre (immersing lower limbs in cool water before exercise) and concurrent cooling methods (applying ice packs or drinking cold beverages during exercise) may help decrease symptom worsening during exercise-induced heat stress in people with MS [67–73]. Our study marks the first attempt to examine the effects of the whole-body concurrent cooling method as therapy for people living with MS having barriers to exercise participation such as walking disability, fatigue, and heat sensitivity. There is an urgent need to develop new therapies and exercise-based rehabilitation treatments that could potentially stabilize or even improve MS symptoms, especially among people who have accumulated substantial disability. In our study, we have demonstrated the feasibility of conducting a progressively intense (moderate to vigorous) aerobic walking training strategy with concurrent cooling (16 °C cool room) using BWST for people with MS requiring ambulatory assistive devices, wheelchairs, and mobility scooters. Eight of the ten participants completed the training, with attendance rates ranging from 80 to 100%. The attendance rate for high intensity aerobic training has been reported to be comparable among individuals with cardiovascular diseases (87%) [74], chronic stroke (85%) [75], and breast cancer (83%) [76]. In order to characterize exercise-induced changes during training [77], we measured physiological responses across training days (Fig. 2). Participants were able to extend the distance walked during each training session (Fig. 1). We noted an exercise-induced increase in tympanic temperature, heart rate, and fatigue; however, mean arterial pressure remained stable during all training sessions (Fig. 2). At the end of the training, our participants experienced improved energy levels measured using SF-36 (36.4%) when compared to the conventional benchmark (11 to 20%) [61] which was higher than that previously reported following robot-assisted gait training (16%) [78]. Furthermore, participants who had greater improvements in fatigue walked faster (Fig. 5a) and also achieved a higher maximal respiratory exchange ratio during GXT after training (Fig. 5b). There were no adverse events (MS relapse, syncope, or medical emergencies) in our cohort, except for fleeting symptoms of neurologic origin, such as pain, pins and needles, and weak legs, which lasted less than 24 h. Whether such fleeting symptoms occurred due to heat (exercise-induced increase in core temperature) sensitivity in our participants is unknown, and further research is required to confirm the physiological mechanisms of worsening MS symptoms during training [79]. It was interesting to note that although participants began their training at 80% of their walking speed, three required manual assistance within just a few minutes during initial sessions, supporting that fatigue and leg weakness are major impediments to effective exercise interventions [80, 81].

### Mode of training and clinically meaningful recovery of gait

We determined whether improvements in walking tests were clinically meaningful. Considering the T25FW test (measured in seconds), we noted that 4 out of 8 participants had clinically meaningful improvements (≥ 20%) after training (Fig. 3a) [57]; similar to those observed in previous studies that evaluated BWST training in people with MS [26, 82]. However, participants in our study walked much slower at baseline (16.4 s on T25FW test) compared to those in previous reports (7.1, and 9.9 s) [26, 82]. When considering fast walking speed measured in cm/s on an instrumented walkway, participants were walking 15.5% faster after 10 weeks of training (14.3 cm/s faster) (Table 3), which is higher than the value determined by Coleman et al. [83] (11 cm/s) to be a clinically meaningful change in people with MS. Considering this benchmark, four participants could be categorized as minimally improved (11 to 17.3 cm/s improvement), one as much improved (17.4 to 22.2 cm/s), and one as very much improved (22.3 cm/s or more) [83]. Furthermore, the gains in fast walking speed were sustained at 3-month follow-up assessment (Table 3), whereas previous examination of robot-assisted gait training showed that training gains (when measured using 10-m walk test) were lost three months later [84]. Lastly, our participants walked considerably faster at self-selected speed overground at 3-month follow up compared to post-training assessment (Table 3). Although there is no consensus as to what value constitutes a clinically meaningful change in self-selected overground walking speed, a change of 12 to 20% in related walking tests is indicative of a meaningful change in MS [59]. Considering this criterion, 6 out of 8 participants made more than 12% improvement on self-selected walking speed post-training, which was sustained in 3 out of 7 participants during 3-month follow up (7.41 cm/sec increase) (Fig. 3b) (Table 3). These findings were in contrast with robot-assisted gait training in which participants had a 7.0 cm/s increase immediately after training but returned to pre levels three months after training [85]. Additionally, spatiotemporal gait parameters at self-selected pace were improved well beyond previous reports employing non-gait specific training methods (legs and trunk resistance training twice a week for eight weeks) [86], supporting that gait quality also improved. Future studies should examine whether similar benefits could be achieved among people who have milder walking impairment or those who have experienced a recent decline due to relapse.

### Ability to perform GXT and improvements in cardiorespiratory reserve

We showed that patients with high levels of disability were able to complete GXT in 10 out of 25 occasions (40% success rate) and 50% of our participants achieved 90% of their age-predicted maximal heart rates during GXT, potentially important information needed for planning of future studies among patients with high levels of MS-related disability [26, 78, 82, 84, 85, 87–92]. We found that our participants with high disability achieved 12.2% greater maximal workload during GXT as a result of training, despite small increases in maximal heart rate (1.8%), maximal respiratory exchange ratio (2.2%), and maximal V̇O_2_ (6.6%). A meaningful change in maximal V̇O_2_ due to an exercise training has been estimated at 0.540 L/min (18.9%) in healthy individuals [93]. Our participants obtained 0.061 L/min increase on average; a 6.6% increase after training, which further increased to 8% at 3-month follow up. We note there is no known clinically meaningful change benchmark for maximal V̇O_2_ applicable to people with MS having severe deconditioning [94]. For patients with severe MS-related disability (EDSS 6.5), the measurement of oxygen uptake efficiency slope is an alternative (sub-maximal) method to express cardiorespiratory fitness when maximal exercise testing is not feasible [62, 63]. We found that the oxygen uptake efficiency slopes were higher in those who had higher maximal V̇O_2_ and maximal workload achieved during GXT (*p* values (not reported), < 0.05) at all three testing time points. As a result, the increase in oxygen uptake efficiency slope during GXT both immediately (12.7%) and 3-month after training (13.5%), could likely be attributed to a combined improvement of cardiovascular, musculoskeletal, and respiratory functions [62, 95]. Future studies should examine the links between improvements in cardiorespiratory reserve, walking speed, and health-related quality of life following training in people with advanced MS [94].

### Improved health-related quality of life

Overall, we noted a clinically meaningful improvement in the quality of life (i.e., more than a 3-point increase in all SF-36 domains except social functioning immediately after training compared to baseline) (Table 3) [64, 65]. Furthermore, improvements in physical functioning, bodily pain, and perception about health compared to last year were significantly improved after training compared to baseline, which was sustained 12 weeks after the intervention ceased (Table 3). When comparing our results to others, robot-assisted gait training of shorter duration (6 weeks, 2 sessions/week) failed to improve SF-36 subcomponents, physical functioning, and bodily pain after training [78]. Likewise, robot-assisted gait training for four weeks (3 sessions/week; 12 sessions) made no change in physical and mental health measured using SF-36 at post, 3-month, and 6-month follow up assessments [84]. Sustained improvement in quality of life (physical functioning), like that observed in our study, suggests that the benefits gained with vigorous cool room BWST training resulted in improved walking ability even after cessation of training, rather than simply short-term performance enhancement, which was meaningful for the participants.

### Vigorously aerobic cool room training might have the potential to affect multiple underlying mechanisms

Aerobic exercise is an intervention that has both neuroprotective and anti-inflammatory benefits [96, 97]. Evidence suggests that progressively intense, aerobic training performed 2 or 3 times per week for at least 8 to 9 weeks could improve walking ability as well as result in a trend towards an increase in resting BDNF levels in people with MS [12] and in other neurological disorders such as stroke [98]. Similarly, our participants with MS experienced statistically significant improvement in walking ability, and 6 out of 7 participants had an increase in resting levels of serum BDNF after training. Further investigation is required to determine whether a simultaneous increase in walking ability and resting serum BDNF levels would result in clinically meaningful restoration of function in MS. With regards to resting serum IL-6 measurements in our study, there were fewer number of data points above minimum detectable limits to glean any meaningful trends (Fig. 5d). However, the improvement in maximal V̇O_2_ was associated with a decrease in resting serum IL-6 levels after training (Fig. 5d). Considering that IL-6 levels are associated with reduced capacity for neuroplasticity [99], further studies examining the effects of training on suppression of inflammation are necessary to understand the molecular mechanisms of recovery.

## Limitations

Despite the fact that this is the first report of vigorous cool room BSWT training among people with progressive MS, there are several limitations to consider. First of all, our study had a very small sample size, thus limiting statistical power to obtain conclusive results. Seven of the 37 potential participants we contacted did not wish to participate in such an exercise program, and two of the 10 participants discontinued the intervention. This suggests that the vigorous cool room treadmill training method is not acceptable to about 20% of people who are eligible. Secondly, we acknowledge that the study was not controlled which tempers the improvements noted in this study [100]. Future studies should include blinded assessors and an active control group, for example, one that includes participants training at lower exercise intensity. Thirdly, as we do not know the characteristics of those who declined to participate in the study, further studies are needed to explore the generalisability of our findings. Lastly, we were unable to complete blood draws in some subjects, and it appeared that hypo-hydration could have been a factor. Although we did not determine whether participants had bladder problems, about 80 to 100% of people with progressive MS have bladder insufficiency [101, 102]. Future trials should consider the issue of hydration during exercise.

## Conclusions

A vigorous cool room walking training is feasible for people with MS using ambulatory assistive devices. We did not identify any adverse events or safety hazards during the training. The total time walked, and distance covered progressively increased while total resting time decreased. People with MS walked significantly faster after cool room training with better gait quality, which was sustained at three months follow up suggesting that there was long-term improvement of function and not simply short-term performance enhancement. Fatigue (SF-36 fatigue/energy/vitality and mFIS), fitness (maximal workload and cardiorespiratory reserve), and quality of life measures (physical functioning, bodily pain, and health compared to last year) improved significantly after training, and improvements on fatigue (mFIS), fitness (maximal workload), and quality of life (physical functioning) were sustained 12 weeks after completion of the program. Vigorous training in a cool room using BWST has the potential to be an effective treatment option for improving walking ability, fatigue, fitness, and quality of life in people with MS using walking aids, which provides a strong rationale for a future clinical trial. There were associations between improvements in walking, fatigue, fitness, and blood biomarkers (serum BDNF and IL-6) that are worthy of further evaluation.

## Supplementary information

**Additional file 1.** Study recruitment flow diagram.

## Data Availability

The data supporting this study are available at request from the corresponding author at the Memorial University of Newfoundland, Canada.

## Abbreviations

°C: Degree celsius
BDNF: Brain-derived neurotrophic factor
BWST: Bodyweight supported treadmill
CNS: Central nervous system
EDSS: Expanded disability status scale
FSS: Fatigue severity scale
GXT: Graded exercise test
HRR: Heart rate reserve
IGF-1: Insulin-like growth factor-1
IL: Interleukin
mFIS: Modified fatigue impact scale
MS: Multiple sclerosis
PAR-Med-X: Physical activity readiness medical examination
PAR-Q: Physical activity readiness questionnaire
r_s_: Spearman’s rank correlation coefficient
SF-36: 36-Item short form
T25FW: Timed 25 foot walk
V̇O_2_: Oxygen consumption
