# Supplementary material for: Vigorous cool room treadmill training to improve walking ability in people with multiple sclerosis who use ambulatory assistive devices: a feasibility study
Source: BMC Neurol. 2020 Jan 22;20:33. doi: 10.1186/s12883-020-1611-0 (PMC6975092; doi:10.1186/s12883-020-1611-0)
Supplement: Supplementary file 1 — Additional file 1. Study recruitment flow diagram. [file 12883_2020_1611_MOESM1_ESM.doc]

**Enrollment**

Assessed for eligibility (n=37)

Excluded (n= 27)

- Declined to participate (n=7)

- Unable to contact (n=7)

- did not receive a call back (n=3)

- unable to reach through telephone or e-mail (n=4)

- Not meeting inclusion criteria (n=13)

- recent fall with injuries, fracture (n=2)

- does not use walking aid (n=6)

- admitted to long term care (n=2)

- moved away (n=1)

- out of town (n=2)

Pre-intervention assessment (n=10)

**Allocation**

Allocated to intervention (n=10)

- Received allocated intervention (n=8)

- Did not receive allocated intervention (n=0)

- Discontinued intervention (n=2)

- fatigue, felt unsafe to drive back home

- started a new MS medication

Post-intervention assessment (n=8)

**Follow-Up**

Lost to follow-up (n=1)

- Unable to contact

Follow-up assessment after 3 months (n=7)

Supplement 1: Study recruitment flow diagram
